# Perspective on Vision Science-Informed Interventions for Central Vision Loss

**DOI:** 10.3389/fnins.2021.734970

**Published:** 2021-11-04

**Authors:** Marcello Maniglia, Kristina M. Visscher, Aaron R. Seitz

**Affiliations:** ^1^Department of Psychology, University of California, Riverside, Riverside, CA, United States; ^2^Department of Neurobiology, The University of Alabama at Birmingham, Birmingham, AL, United States

**Keywords:** visual rehabilitation, perceptual learning, macular degeneration, oculomotor abilities, neural plasticity, clinical intervention strategy perspective

## Abstract

Pathologies affecting central vision, and macular degeneration (MD) in particular, represent a growing health concern worldwide, and the leading cause of blindness in the Western World. To cope with the loss of central vision, MD patients often develop compensatory strategies, such as the adoption of a Preferred Retinal Locus (PRL), which they use as a substitute fovea. However, visual acuity and fixation stability in the visual periphery are poorer, leaving many MD patients struggling with tasks such as reading and recognizing faces. Current non-invasive rehabilitative interventions are usually of two types: *oculomotor*, aiming at training eye movements or teaching patients to use or develop a PRL, or *perceptual*, with the goal of improving visual abilities in the PRL. These training protocols are usually tested over a series of outcome assessments mainly measuring low-level visual abilities (visual acuity, contrast sensitivity) and reading. However, extant approaches lead to mixed success, and in general have exhibited large individual differences. Recent breakthroughs in vision science have shown that loss of central vision affects not only low-level visual abilities and oculomotor mechanisms, but also higher-level attentional and cognitive processes. We suggest that effective interventions for rehabilitation after central vision loss should then not only integrate low-level vision and oculomotor training, but also take into account higher level attentional and cognitive mechanisms.

## Introduction

With the growing elderly population in the United States and worldwide, age-related diseases are becoming a serious health issue that demands increasingly high healthcare expenditures (agingstats.gov). Among these conditions, pathologies affecting vision are particularly serious, with macular degeneration (MD), one of the most common causes of vision loss, projected to affect 248 million people worldwide by 2040 (Wong et al., 2014). Given its crucial role among human senses, consequences of loss of central vision are manifold, and affect visual abilities, eye movement strategies and even cognitive networks.

In this paper, we briefly review current non-invasive rehabilitative interventions for central vision loss (focusing on MD), in particular those within the fields of vision science and optometry, discuss strengths and limitations, and propose a new approach to rehabilitation and assessment in low vision that integrates insights from both fields.

## Macular Degeneration: An Overview

Macular degeneration is characterized by damage to photoreceptors in the center of the visual field, which in turn has detrimental effects on daily tasks such as navigating, reading, and recognizing faces. MD can be of two types, wet (exudative) or dry (geographic atrophy) (Ferris et al., 1984; Zarbin, 2004; de Jong, 2006). Wet MD is usually caused by abnormal blood vessels leaking fluid into the foveal region of the retina, it has a more abrupt onset than dry MD and patients often experience sudden loss of central vision. Dry MD is a congenital pathology, which usually manifests with retinal scotomas (blind portions of the visual field) progressively leading to central vision loss. While initial symptoms are usually limited to distortions of the retinal grid and shadows (Simunovic, 2015), late stage MD results in a large dense central scotoma that encompasses the fovea and the perifovea, extending up to 20° in the periphery (Cheung and Legge, 2005). Consequences of MD are large-scale, and involve difficulties in reading (Bullimore and Bailey, 1995), recognizing faces (Bernard and Chung, 2016), driving (Bowers et al., 2005) eventually taking a toll on the quality of life of these individuals (Šiaudvytyte et al., 2012).

Macular degeneration patients tend to spontaneously develop compensatory strategies to cope with the loss of central vision. A common strategy observed in this clinical population is the adoption of a peripheral retinal region in substitution of the fovea, usually referred to as the preferred retinal locus (PRL) (von Noorden and Mackensen, 1962; Fletcher and Schuchard, 1997). This retinal spot effectively takes over duties of the fovea as a locus of fixation and, in some cases, oculomotor reference (White and Bedell, 1990; Whittaker and Cummings, 1990). However, not all patients manage to develop a PRL, effective use of PRLs varies across patients, and the mechanisms of PRL development are still not clear. While evidence from the simulated scotoma literature suggests that peripheral retinal regions with higher attentional capabilities might be good candidates for developing a PRL (Barraza-Bernal et al., 2017), no definitive evidence of this mechanism exists in the clinical literature. It seems that several aspects of residual vision play a role in determining PRL formation, including: visual acuity, residual size of the visual field, dimension of the scotoma, proximity of the fovea, visual task and light conditions (Lei and Schuchard, 1997; Duret et al., 1999; Safran et al., 1999; Altpeter et al., 2000; Déruaz et al., 2002). In some cases, different PRLs are used by the same patient depending on the tasks demands (Lei and Schuchard, 1997; Sullivan et al., 2008; Crossland et al., 2011), with fine acuity judgments placing different demands on the visual periphery than tasks such as face recognition that involve integration across multiple features. Moreover, even when patients do develop a PRL, they still struggle with numerous visual abilities which are naturally reduced in the periphery, including: contrast sensitivity, orientation discrimination, visual acuity (Johnson et al., 1978), word identification speed (Latham and Whitaker, 1996), among others. These functional properties come about at least in part because of structural and anatomical limitations of peripheral vision (Strasburger et al., 2011), which constrains vision quality within the PRL.

Additionally, loss of central vision not only irreversibly compromises the retinal location with the highest visual resolution, but also deprives the visual system of its oculomotor reference for planning and executing saccades (White and Bedell, 1990; Whittaker and Cummings, 1990). While some patients spontaneously learn to use the PRL as their reference for ballistic eye movements, in a process called “saccadic re-referencing” (White and Bedell, 1990), this is not always the case and often patients experience partial or no re-referencing (Crossland et al., 2005). The process of re-referencing seems to be further complicated by differences in oculomotor behavior between volitional and automatic saccades (Chung, 2013b), with some participants showing re-referencing for one type but not the other (White and Bedell, 1990). Even in cases of complete saccadic re-referencing toward the PRL, the reduced fixation stability (Culham et al., 1993; Crossland et al., 2005) and eye movement control (Timberlake et al., 1986; Whittaker et al., 1991) of the periphery, limit eccentric vision.

Further, patients with MD are usually elderly, a population characterized by generally reduced visual functions, including contrast sensitivity (Richards, 1977; Derefeldt et al., 1979), visual acuity (Chapanis, 1950; Kahn et al., 1977), and orientation discrimination (Betts et al., 2007). In general, vision with the PRL presents several challenges for the patients and currently the mechanisms and time course of its development are still unclear: there is evidence suggesting that the PRL is not necessarily the most sensitive portion of the spared retina (Fine and Rubin, 1999; Petre et al., 2000); that it might take months to develop and its location can change over time (Crossland et al., 2005), or that some patients never develop one (Fletcher and Schuchard, 1997).

Crucially, loss of central vision might bear consequences reaching far beyond perceptual and oculomotor brain regions. When patients develop compensatory oculomotor strategies to bypass the foveal reference, these are accompanied by concomitant changes in attentional deployment strategies. For example, the fact that objects attracting attention now require eye movements to bring them to the PRL rather than the fovea means that attention processes need to be remapped to incorporate the PRL. Consistent with this, neuroimaging data shows that learning to use peripheral vision requires that participants with MD integrate their PRL with cognitive control networks (Sabbah et al., 2017).

Consequences of central vision loss are dramatic for the patients’ quality of life and there still is a lot to be understood concerning the mechanisms behind the spontaneous compensatory strategies that patients adopt, whose variety seems reflected in studies with simulated scotoma as well (Maniglia et al., 2020a), a recently adopted paradigm which may help in developing targeted rehabilitative interventions. In regard to treating MD, while invasive approaches (e.g., intravitreal anti-vascular endothelial growth factor agents, Ammar et al., 2020) are used to counteract the effects of the exudative form of MD (Wet AMD), no current standard intervention exists for the progressive type of MD (dry MD), which accounts for 85% of the cases (Jager et al., 2008). To date, non-invasive approaches to low vision rehabilitation come from the neighboring fields of vision science and optometry and are generally of two types: oculomotor and perceptual. These approaches adopt different theoretical frameworks and focus on different aspects of the rehabilitative path: oculomotor approaches teach patients to improve their eye movement control and coordination, sometimes by training them to use a different (and more appropriate) peripheral retinal spot in substitution of their spontaneously developed PRL (Nilsson, 1990; Nilsson et al., 2003; Verdina et al., 2013, 2020; Morales et al., 2020), while perceptual interventions focus on alleviating the limitations of peripheral vision by improving general peripheral visual abilities or vision within the PRL (Chung, 2011; Plank et al., 2014; Tarita-Nistor et al., 2014; Maniglia et al., 2016, 2020b). While there exist other types of intervention, such as the use of low vision aids and magnification devices (Bailey, 1987; Chung and Johnston, 1989; Markowitz, 2006), which often represent the first line of intervention in MD rehabilitation, these find limited use because of their limited practical use in activities of daily life such as dressing an eating (Jeong and Moon, 2011). In the following sections, we discuss strengths and limitations of *oculomotor* and *perceptual* approaches to central vision loss rehabilitation.

## Oculomotor Interventions

Research on oculomotor interventions finds improvements in reading speed, fixation stability and visual acuity when MD patients are trained with eye tracking technology (Nilsson et al., 2003; Verdina et al., 2013, 2020; Morales et al., 2015, 2020) or computerized programs (Nilsson et al., 2003; Coco-Martin et al., 2013; Rosengarth et al., 2013) to improve muscle control, eye movement coordination and fixation stability. These approaches are usually conducted under the constant supervision of therapists and in some cases involve the use of magnification devices.

An interesting approach involves teaching MD patients to use a different peripheral retinal spot [trained retinal locus (TRL)] in lieu of their PRL located in a more convenient portion of the spared peripheral retina (Nilsson, 1990; Nilsson et al., 2003; Estudillo et al., 2017; Morales et al., 2020). The TRL is usually chosen to be in an area large enough to accommodate multiple characters (to allow reading) and with a relatively high visual acuity (to allow letter recognition). In a retrospective study on MD patients who were trained with the TRL protocol from the Macular Integrity Assessment (MAIA) microperimetry, Estudillo et al. (2017) reported improvement in monocular fixation stability, reading speed, and visual acuity, measured 1 week after training was completed. Daibert-Nido et al. (2019), in a similar retrospective study on a cohort of patients with different retinal pathologies, reported visual acuity gain after training in MD patients. All participants trained with a TRL showed a shift in PRL location toward the superior quadrant of the retina. Similarly, Morales et al. (2020) and Sahli et al. (2020) using microperimetry-informed biofeedback fixation training to teach patients to use a more convenient retinal location for fixation, reported improvement in fixation stability, reading acuity, reading speed and in self-reported visual functions (as assessed by the VFQ-25).

However, a limitation of oculomotor approaches is that the mechanisms of neural plasticity associated with these clinical approaches are not well studied. To date, only one neuroimaging study investigated neural plasticity changes associated with eye movement training (Rosengarth et al., 2013). In this study, no significant changes in blood-oxygen-level-dependent (BOLD) signal were observed in early visual (V1, V2, and V3) or higher-level associative areas (LOC, fusiform gyrus, ITG), however a positive correlation was found between changes in fixation stability and changes in brain activation in these areas after the first stage of training. Additionally, except for recently introduced automatized programs (e.g., biofeedback training from MAIA, see Nilsson, 1990; Nilsson et al., 2003; Estudillo et al., 2017; Morales et al., 2020), these interventions seem to vary greatly and rely on the expertise and subjective approach of each therapist. Additionally, trial studies using these techniques are sometimes lacking robustness, which hinders a proper evaluation of efficacy (Gaffney et al., 2014). In general, optometry training requires highly specialized personnel and, in some cases, expensive equipment (i.e., microperimetry devices).

## Perceptual Interventions

Approaches focusing on perceptual interventions commonly stem from the field of Perceptual learning (PL), which examines how perceptual skills improve following extensive task practice, in a process mediated by neural plasticity (Sagi, 2011). Collectively PL research shows that visual training on simple tasks of orientation discrimination, contrast detection and letter recognition can improve visual acuity, contrast sensitivity and reduce visual crowding in the PRL of MD patients (Chung, 2011; Plank et al., 2014; Maniglia et al., 2016, 2020b). The rationale behind PL approaches is that training on basic visual features can induce beneficial neural plasticity in early visual areas, which in turn improves visual processing at later stages (Polat, 2009), leading to, in at least some cases, generalization of learning from the trained task to other visual abilities that may share common neural substrates. This concept holds great promise for rehabilitation after MD, since it involves improvements in visual processing that do not depend on the retina and its integrity.

For example, two studies (Chung, 2011; Tarita-Nistor et al., 2014) trained MD participants using a Rapid Serial Visual Presentation (RSVP) paradigm, in which a series of visual stimuli (in this case words) were briefly presented on a screen and participants were asked to identify them. Results showed improvements in the trained task in both studies, with some evidence that learning can generalize to reading acuity and maximum reading speed (Tarita-Nistor et al., 2014). These learning gains are thought to be due to the use of near-threshold stimuli, which can promote both learning gain (Tsodyks and Gilbert, 2004; Seitz and Watanabe, 2005; Sagi, 2011) and transfer (Polat, 2009). However, Plank et al. (2014) trained MD patients with a classic texture detection paradigm (Karni and Sagi, 1991), reporting improvements in the trained task but limited transfer of learning beyond the specifics of the trained task (although there was some transfer to Vernier Acuity). This may be because the task used has shown high orientation and spatial location specificity in previous research (Karni and Sagi, 1991). More recently Maniglia et al. (2016; 2020b) trained MD patients with a lateral masking configuration similar to one previously used to treat amblyopia (Polat et al., 2004) and myopia (Tan and Fong, 2008), a protocol that has shown to promote generalization of learning in mild visual pathologies (Polat, 2009) and healthy peripheral vision (Maniglia et al., 2011). Results showed that patients improved in untrained visual abilities such as contrast sensitivity function and visual acuity; additionally, when the lateral masking training was preceded by a training to improve fixation stability, usually poor in the MD population (Macedo et al., 2011), this training led to reduction of crowding and improved reading speed (Maniglia et al., 2020b). Overall, these studies suggest potential of perceptual learning based methodologies as potential methodologies to improve vision after central vision loss.

However, while promising, to date perceptual learning paradigms have shown only moderate effectiveness in MD, in particular in producing learning generalization (Chung, 2011; Plank et al., 2014; Maniglia et al., 2016, 2020b), which is a fundamental outcome for clinically relevant intervention. A possible reason is that most perceptual learning approaches were originally developed to train vision in those with intact central vision and do not necessarily address the particular needs of the visual system after central vision loss. For example, theoretical frameworks underlying perceptual learning often target brain plasticity to overcome optical limitations (myopia, presbyopia) or neural atypicalities (amblyopia) in clinical populations. In these studies, participants are trained on simple perceptual tasks such as orientation discrimination or contrast threshold and achieve a post-training visual performance that is comparable to that of individuals with a healthy visual system (Tan and Fong, 2008). In the context of MD, in which central vision loss cannot be reverted, current perceptual learning approaches have focused on improving basic visual functions in a peripheral region (the PRL) to be more like performance in central vision, thus improving visual acuity, contrast sensitivity or visual crowding (Chung, 2011; Plank et al., 2014; Maniglia et al., 2016, 2020b). A common limitation of these studies is that, with some exceptions (e.g., Chung, 2011), the monitoring of eye movements is either conducted in the absence of concomitant fundus imaging, which may affect the accuracy of the calibration (Rosengarth et al., 2013; Plank et al., 2014) or eye tracking is replaced by visual aids (Astle et al., 2015; Maniglia et al., 2020b) leading to a less precise retinal localization.

This suggests a fundamental difference between what classic studies of perceptual learning aim to achieve in pathologies in which the photoreceptors are not strongly affected (e.g., amblyopia, presbyopia, and myopia) vs. in MD where central vision is lost. After central vision loss, the goal of a rehabilitative approach is not only to improve peripheral vision, but it is also necessary to re-reference the periphery so that eye movements are now directed to peripheral locations rather than the fovea, which is now non-functional. The anatomical network that originally had one role (the functions of peripheral vision) must be modified so that it performs a different role (the functions of central vision), and this can happen through training-induced neural plasticity.

Thus perceptual learning in MD needs to be targeted to induce a different form of plasticity than that of standard perceptual learning (SPL) paradigms, potentially at a larger scale than in amblyopia, presbyopia or myopia. It follows that adopting training paradigms designed to improve the “visual performance” in the PRL might only be targeting one component of what is needed to improve ecological vision in the PRL: Neural plasticity has to use what is structurally available and reroute it to improve residual vision, an effort that requires larger-scale plasticity (beyond sensory areas) with respect to SPL and involves multiple brain regions and networks. Recent manipulations of classic PL paradigms, involving exogenous attention (Szpiro and Carrasco, 2015), concomitant brain stimulation (Camilleri et al., 2014; Contemori et al., 2019), and training two different tasks (e.g., orientation discrimination and contrast detection) within the same training regime (Xiao et al., 2008), which showed larger learning and training effects, might provide promising templates to be successfully implemented in the treatment of MD.

Still, to develop an effective rehabilitative intervention in the context of central vision loss, it is necessary incorporate into the training the larger-scale consequences of central vision loss that go beyond sensory and oculomotor areas and also considers plasticity in cognitive control networks.

## Potential Role for Cognitive Control Interventions

After binocular central vision loss, the normal locus of fixation and target of eye movements (the fovea) is no longer viable. Thus, MD patients must learn to perform visual tasks using a peripheral portion of the retina. This requires orienting and stabilizing eye movements toward that peripheral location, but also requires learning to attend to peripheral vision.

Spatial attention is a critical component of vision that allows improvements in processing of selected stimuli (Carrasco, 2011). Normally sighted individuals attend to central vision the majority of the time. Those with central vision loss must learn to attend instead to the periphery. Thus, attention networks that usually help process information in central vision must now direct resources to peripheral vision. Although studies of PL typically focus on learning stimulus features, research shows that attention and cognitive control (Ahissar and Hochstein, 1993; Schoups et al., 2001; Byers and Serences, 2012; Bays et al., 2015; Szpiro and Carrasco, 2015) play key roles in PL. Studies using the simulated scotoma paradigm showed improved performance on an attention task preferentially in a trained PRL vs. untrained locations (Liu and Kwon, 2016) and that normally sighted individuals can learn to maintain stable fixation at a PRL when central vision is occluded (Kwon et al., 2013).

Attention and eye movements are inextricably linked, with eye movements to a visual field location referred to as “overt attention” (Moore, 2001). The ability to easily focus spared vision onto peripheral objects, and to make eye movements so spared vision (the PRL) is “re-referenced” to land and stay stable on these objects is one of the most fundamental aspects of visual performance changes after central vision loss, and a common clinical target for MD (Frennesson et al., 1995; Seiple et al., 2005; Pijnacker et al., 2011). However, most PL studies do not address eye movements or spatial attention more generally, and instead discourage eye movements and require maintained focus to the same location throughout training (e.g., RSVP paradigms).

Most natural vision tasks jointly involve visual sensitivity, spatial integration and eye movements. Although a complete dissociation of these is not possible, and other candidate domains could be considered, these three domains are well documented to be important for PL.

## Spontaneous Plasticity After Macular Degeneration

There is robust evidence of spontaneous neural plasticity changes in individuals with central vision loss even before taking part in vision training studies or rehabilitation interventions (Baker et al., 2005; Dilks et al., 2009, 2014; Chung, 2013a; Haun and Peli, 2015; Maniglia et al., 2018). In particular, neuroimaging studies suggest that early visual cortex undergoes spontaneous reorganization of varying degrees depending on factors like the extent of residual foveal vision (Baker et al., 2005; Dilks et al., 2009, 2014) and type of task (Masuda et al., 2008; Wandell and Smirnakis, 2009; Baseler et al., 2011), however it is still debated whether this neuroimaging evidence reflects a real remapping of early visual cortex (Baker et al., 2005; Dilks et al., 2009, 2014) or is due to attentional/higher-level feedback (Masuda et al., 2008; Wandell and Smirnakis, 2009).

Similarly, psychophysical studies in MD patients suggest that spontaneous cortical reorganization might be responsible for observed changes in the crowding zone around the PRL (Chung, 2013a). Crowding, the inability to identify a target when presented embedded between similar elements (Levi, 2008), has a characteristic radial-tangential anisotropy that, while almost negligible in the fovea, increases with eccentricity. Chung (2013a) showed that such radial asymmetry, which would be expected in a peripheral retinal location like the PRL, was almost absent in MD patients, suggesting a fovea-like reorganization in the PRL. Interestingly, the change in the shape of the crowding zone reported by Chung (2013a) is consistent with that observed in individuals trained with artificial scotomas (Chen et al., 2019). Maniglia et al. (2018) observed that MD patients showed reduced lateral inhibition in their PRL with respect to healthy participants tested at the same eccentricity. Lateral inhibition is usually considered an early visual cortex phenomenon (Polat et al., 1998) and previous studies showed that it decreases with visual training (Polat and Sagi, 1994; Maniglia et al., 2011). These findings suggest that MD patients might have reduced their lateral inhibition around the PRL through use-dependent plasticity.

In general, this spontaneous reorganization after retinal lesion might also be responsible for some of the differences observed in perceptual and training effects in patients with central vision loss with respect to healthy participants (Van der Stigchel et al., 2013; Haun and Peli, 2015). Plank et al. (2014) investigated neural plasticity changes in MD patients after PL training. They used a texture discrimination task and reported that, in those participants with good fixation stability, visual stimulus-driven BOLD signal in early visual cortex increased with training. This work suggests that neural processing of the visual stimuli in perceptual areas of the MD brain is influenced by training.

Other work supports the hypothesis that learning to use peripheral vision involves not only perceptual and oculomotor systems, but also integrating cognitive control with vision. Work in both patients with MD and controls suggests that visual field-specific variation in sustained attention influences choice of a PRL (Altpeter et al., 2000; Barraza-Bernal et al., 2017). Studies in healthy participants showed that cortical regions processing central vision are preferentially connected to fronto-parietal brain regions associated with attention (Griffis et al., 2017; Sims et al., 2021) with respect to regions processing peripheral vision, which is consistent with stronger top-down control of central vision (Chen and Treisman, 2008; Zhaoping, 2017).

Therefore, being forced to shift to a non-foveal reference has larger consequences than just reduced visual acuity: the visual system must change its default strategies of orienting attention (and consequently eye movements) from the fovea to the PRL. Neuroimaging data further supports the hypothesis that learning to use peripheral vision requires that participants with MD integrate their PRL with cognitive control networks. Recent evidence shows that loss of central vision affects connectivity to parietal brain regions associated with attention and cognitive control (Sabbah et al., 2017). Together these data show that learning to use peripheral vision after MD also involves cognitive control and attention.

Further, data from healthy controls show that functional connectivity between early visual areas and areas associated with attention/cognitive control (Altpeter et al., 2000; Li et al., 2004; Dosenbach et al., 2008) are stronger in representations of central than peripheral vision (Griffis et al., 2015; Sims et al., 2021). While extant literature has not explicitly addressed PL-based reorganization of attention networks after central vision loss, there is substantial evidence that attention processes are highly plastic (Frankó et al., 2010; Byers and Serences, 2012; Bays et al., 2015), and that attentional processes are altered in MD (Baker et al., 2005; Masuda et al., 2008; Dilks et al., 2009).

Contrary to the ecological learning conditions of MD, the majority of perceptual learning approaches involve maintaining attention at the same location throughout the task and typically discourage eye movements (Chung, 2011; Plank et al., 2014; Tarita-Nistor et al., 2014; Maniglia et al., 2016, 2020b). Thus there is a wide gap between the mechanisms that are the typical targets of the perceptual and oculomotor approaches, where they rely upon different theoretical foundations (sensory improvement in the former and eye movement control in the latter), but also likely impact different brain networks (eye movement and cognitive control networks in the former, and early visual cortex in the latter). This may explain why each has mixed success; their efficacy may depend upon which visual processes individual patients have the greatest need for training. To date there has been little mix between the two approaches. For example, a recent study combined a simple fixation stability training with perceptual learning training on contrast sensitivity in MD, without recording eye movements (Maniglia et al., 2020b) while another study, using the simulated scotoma framework (see next section for details), combined perceptual learning with oculomotor training (Liu and Kwon, 2016). However, to date, no rehabilitative intervention has systematically compared oculomotor training and low-level perceptual learning or combined the two in patients with central vision loss.

## Simulated Scotoma as a Model System for Macular Degeneration

Recently, a new approach has been developed that uses eye tracking-based simulation of central vision loss in normally seeing individuals as a lab-based model system to study vision in the presence of a central scotoma, in particular oculomotor strategies, PRL development, and clinical interventions in controlled environment (Bertera, 1988; Fine and Rubin, 1999; Pidcoe and Wetze, 2006; Aguilar and Castet, 2011; Kwon et al., 2013; Walsh and Liu, 2014; Liu and Kwon, 2016; Barraza-Bernal et al., 2017a,b; Chen et al., 2019; Maniglia et al., 2019, 2020a; Costela et al., 2020; Xie et al., 2020). In this framework, an opaque occluder of a few degrees radius is generated and controlled by a computer connected to an eye tracker to obstruct central vision in real time. This approach has been used in a large number of studies, which have made clear that the paradigm can influence participants’ visual experiences (Kwon et al., 2013; Walsh and Liu, 2014; Barraza-Bernal et al., 2017a,b; Chen et al., 2019; Maniglia et al., 2020a). This paradigm addresses issues of recruitment and compliance that often characterize clinical research, thus offering a promising alternative to the direct involvement of MD patients.

**FIGURE 1:**
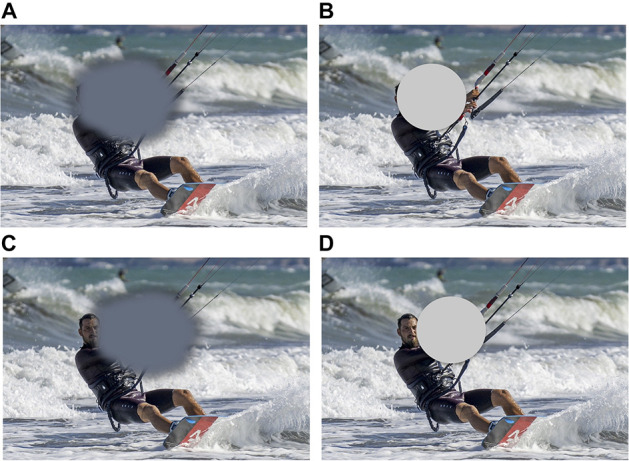
**(A)** Typical representation of the visual field of a patient with central vision loss. In reality, patients with central vision loss are rarely aware of the location and extent of their scotoma, and, crucially, said scotoma does not present clear-cut borders as it is the case of the artificial scotoma, which is shown in panel **(B)**. A typical compensatory strategy in patients with central vision loss is the development of a peripheral retinal locus, or PRL, an eccentric fixation region close to the border of the scotoma, that patients use to fixate and solve demanding visual tasks, i.e., face recognition. Panel **(C)** shows where the scotoma might be placed to allow the face in the image to fall outside the scotoma and on the PRL. Panel **(D)** shows placement of a simulated scotoma allowing the face to be visible. Note once more that the hard edges of the simulated scotoma are different from the true scotoma, and may contribute to speeded development of a PRL with this paradigm (Walsh and Liu, 2014).

In particular, simulating central vision loss might shed light on the mechanisms underling eye movement strategies and PRL development in the MD population (Crossland et al., 2005). A better understanding of such strategies may allow for the development of individualized training, which can be tailored to each patient’s oculomotor profile or, conversely, could identify more effective oculomotor strategies that could be taught to patients. Figure 1 shows a typical example of a simulated scotoma. While they share many similarities with scotomas found in MD, a key difference (beyond that simulated scotomas are studied in otherwise visually healthy individuals) is that simulated scotomas are often more regular than real scotomas and present an explicit obstruction of central vision, unlike pathological scotomas that nearly always lead to visual filling-in of the blind spot(s).

The simulated scotoma model is increasingly being used to test training protocols with potential rehabilitative applications. For example, Kwon et al. (2013) showed that healthy participants trained with a visual search protocol incorporating the simulated scotoma developed oculomotor strategies similar to those observed in MD patients. Namely, participants developed a PRL for fixation and re-referenced saccades toward that PRL. Walsh and Liu (2014) showed that participants trained with a similar paradigm (but with an “invisible” scotoma, i.e., an occluder of the same color as the screen’s background), would most commonly develop a single PRL, as observed in patients. This was despite the lack of an incentive to select and consolidate a single retinal location, and was in fact less efficient than using multiple, functionally comparable peripheral retinal locations (Xie et al., 2020). In other words, healthy vision participants tend to develop a PRL similar to that observed in MD patients. Interestingly, Barraza-Bernal et al. (2017), in another training study using simulated scotoma, showed that locations in the peripheral visual field with high attentional capabilities are likely candidates for PRL development. Following these studies that focused on oculomotor behavior, others showed that visual training in conditions of simulated central vision loss leads to improvement in both oculomotor and perceptual functions, such as fixation stability, saccadic re-referencing toward the PRL (Maniglia et al., 2020a) peripheral visual acuity (Maniglia et al., 2020a) and reading (Liu and Kwon, 2016).

However, there are several differences between pathological and simulated scotomas. As examples, simulated scotomas are typically uniform across time and have visible boundaries, which may be used as an oculomotor reference to redirect saccades (Van der Stigchel et al., 2013; Walsh and Liu, 2014), and those with simulated scotomas experience central vision loss for a short period a day and for just a few handfuls of days. On the other hand, in MD, the size and shape of scotomas change progressively across time, patients are typically unaware of their boundaries (Safran and Landis, 1999; Fletcher et al., 2012), and studied patients often have years of experience with full-time central vision loss (Crossland et al., 2005). Also, notably, PRL development is often slow for MD patients (Crossland et al., 2005) while it seems to be much faster in the case of simulated scotoma (Kwon et al., 2013), perhaps due to the visible boundaries of the occluder (Walsh and Liu, 2014).

Indeed, patients with MD are typically unaware of the size, location or even existence of their scotoma (Safran and Landis, 1999; Fletcher et al., 2012) and often struggle for months to develop a PRL (Crossland et al., 2005). Still, qualitative similarities between studies of peripheral looking strategies in patients with MD and research participants with simulated scotomas abound, including recent evidence that healthy participants trained with asymmetrical scotoma sizes maintain the PRL of the eye with the smaller scotoma (Lei and Chung, 2020), or that they maintain constancy of PRL location and oculomotor characteristics across tasks (Barraza-Bernal et al., 2017; Maniglia et al., 2020a) or scotoma size (Costela et al., 2020), all of which can be observed in clinical reports of numerous MD patients.

## Toward an Integrated Intervention for Central Vision Loss

**FIGURE 2:**
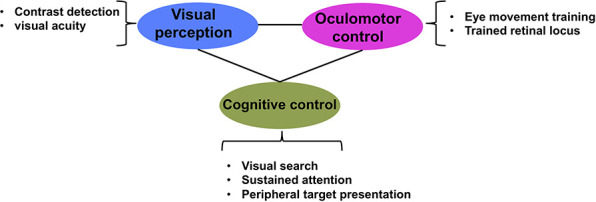
Multidimensional approach to low vision rehabilitation in MD. Figure illustrates how each of visual perception, oculomotor control and cognitive control are interconnected dimensions of vision that contribute to effective vision.

Our central premise is that translational approaches to rehabilitation in individuals with MD can be advanced by approaching intervention in a coordinated way that takes into account eye movement planning, cognitive control mechanisms and perceptual training. This more holistic intervention approach for patients with central vision loss takes into account the multitude of systems and networks affected by loss of central vision (Figure 2). This figure illustrates our proposal that an effective intervention should jointly operate on all the levels that are compromised by the loss of central vision, namely low-level vision, oculomotor control and cognitive control. Specifically, low-level vision could be improved by training contrast detection and visual acuity, similar to what current PL paradigms do (Chung, 2011; Plank et al., 2014; Tarita-Nistor et al., 2014; Maniglia et al., 2016, 2020b); oculomotor control can be trained with eye tracking technology (Nilsson et al., 2003; Verdina et al., 2013, 2020; Morales et al., 2015, 2020) or computerized programs (Nilsson et al., 2003; Coco-Martin et al., 2013; Rosengarth et al., 2013); and cognitive control can be trained with visual search and/or tasks engaging various components of attention. Such an approach takes into account the large-scale consequences of central vision loss, rather than focusing on single aspects or systems.

We tested a prototype of the integrated paradigm, called *coordinated attentional training* (CAT), where we combined classic low-level vision and oculomotor approaches from vision science and optometry interventions (perceptual improvement and oculomotor coordination), with elements that explicitly target higher level, cognitive control networks. To do so, CAT utilized a contrast detection task with random stimulus presentation and visuo-acoustic cues (see below for details). In order to successfully complete the task, the participant is required to: (i) maintain vigilance for relatively long periods, (ii) detect objects in the near periphery, (iii) orient attention to objects quickly, and (iv) move areas of spared vision (outside of the artificial scotoma) to those locations. These functions are associated with cingulo-opercular and fronto-parietal brain network (Altpeter et al., 2000; Li et al., 2004; Dosenbach et al., 2008).

## Preliminary Tests of Integrated Intervention

**FIGURE 3:**
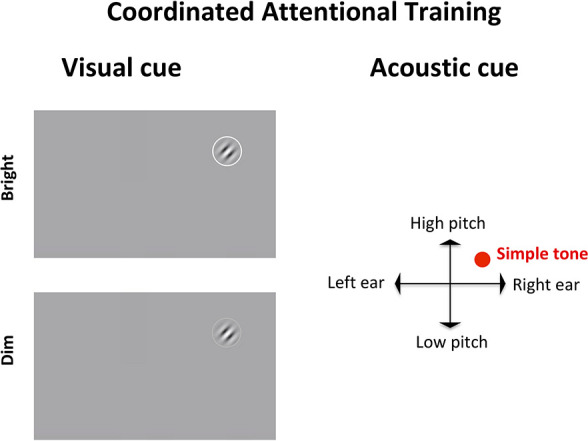
Coordinated attentional training (CAT) display. The target, a Gabor patch tilted left or right, appears in a random location on screen, accompanied by a visual (left) and an acoustic (right) cue. The visual cue, a circle surrounding the target, is either bright (100% luminance) or dim (60% luminance). The auditory cue is a simple tone whose pitch is mapped to the *y* axis of the target (lower for the bottom of the screen, higher for the top) and whose left-right panning target (based upon inter-aural time/level differences) depends on the target location on the *x* axis.

As proof of principle, we report early results from two studies, one conducted with healthy participants trained with simulated scotoma and the other with MD patients. 19 healthy participants for the simulated scotoma study (20.4 ± 1.8 years, 12 females) and 11 MD patients (62.6 ± 15.8 years, 6 females) were randomly assigned between *CAT* (10 healthy participants and 6 MD patients, see Figure 3) *or a SPL* approach (9 healthy participants and 5 MD patients). In the MD study, patients were selected according to the following inclusion criteria: diagnosis of binocular dry MD with central scotoma >2 degrees with record of stability >2 years. All MD had received some form of visual rehabilitation before the study, albeit none of them took part in a perceptual learning study. We note that participants are not age-matched between these two studies. While it is conventional to use younger participants in research using simulated scotomas (e.g., Kwon et al., 2013; Liu and Kwon, 2016; Barraza-Bernal et al., 2017), this does limit direct comparisons between the two groups.

In SPL, the target, a Gabor patch, appeared always in the center of the screen, and was coupled with a neutral acoustic cue. In CAT, the target could appear anywhere on screen, requiring a search and re-orienting of gaze toward the target. The target was accompanied by a visual cue (a circle around the target) that was either bright (100% luminance) or dim (60% luminance), meaning that on some trials the target would be located via parallel search while in others it would require serial search. Additionally, the target in CAT was accompanied by an auditory cue indicating its position on screen. Specifically, the auditory cue would be panned left or right according to the horizontal position of the target (based upon inter-aural time/level differences) and its pitch would be higher or lower depending on the target position along the vertical axis. Thus, while SPL involved a more standard, static perceptual learning paradigm, CAT incorporated shifts of attention toward different (multisensorily) cued locations in space.

The training regime was composed by 10 daily sessions of 500 trials. In both types of training, contrast and spatial frequency of the Gabor patch were subject to a staircase procedure: specifically, the contrast started from 20% and progressively decreased following correct responses according to a 3:1 staircase (after 3 correct responses, the contrast was decreased by, or after 1 incorrect response increased by, 0.1 log units). Once a 1% contrast was reached, the spatial frequency (that started at 3 cycles per degrees [cpd]) would double, while the contrast would reset to 20%. Participants received auditory feedback on their performance. During training, the target was always visible and remained on screen until the participant’s response. Participants were instructed to report the orientation of the target as quickly and accurately as possible. Each training session lasted 500 trials (∼45 min). We note that during training we did not enforce fixation either in patients or those with a simulated scotoma so that they would have opportunity to “discover” and then train what peripheral looking strategies might work best for them. Participants were tested on a series of assessment tasks aimed at measuring both low-level, perceptual functions (i.e., contrast sensitivity, visual acuity, visual crowding), and mid- and higher-level visual and cognitive functions (i.e., motion detection, reading speed and acuity, and functional attention as assessed with the Trail Making Test). For both healthy participants and MD patients, viewing was binocular during training and assessments.

In the simulated scotoma study, an opaque disk of 75% luminance and 10 degrees diameter was presented at all times in the center of the visual field of the participants, rendered in real time by a gaze-contingent protocol based on eye positions recorded by a high-sampling rate eye tracker (Eyelink 1000, 500 Hz), which was used to render the scotoma with minimal latency (28 ms, median value of 50 measurements, corresponding to three frames in the worst-case scenario).

While the sample size is small, we note that there is some evidence of statistically reliable gains from the CAT paradigm. Repeated measure ANOVA with Time and Group as factors showed a significant main effect of training on visual acuity in the MD group (*F*(1,9) = 7.067, *p* = 0.026, η^2^ = 0.005, Bayes factor = 2.826). Paired *t*-tests within each group showed significant training effect for the CAT group (*t*(5) = 2.654, *p* = 0.023), but not SPL (*t*(4) = 1.173, *p* = 0.153). A repeated measure ANOVA conducted on visual acuity measured during an oculomotor task [not shown here, see Maniglia et al. (2020a) for details] also showed a significant main effect of training (*F*(1,17) = 7.265, *p* = 0.015, η^2^ = 0.168, Bayes factor = 7.284). We also observed trends for No statistical comparison looking at CAT vs. SPL (interaction of Group × Time) reached significance (all *p* > 0.05). Bayes factor for the interaction Group × Time was 1.475 for Visual Acuity in the MD group, indicating weak to moderate evidence for H1 (CAT > SPL). All the remaining Bayes factor were >1/3 and <1, indicating anecdotal evidence for either H0 or H1. All statistical comparisons are reported in the Supplementary Material.

**FIGURE 4:**
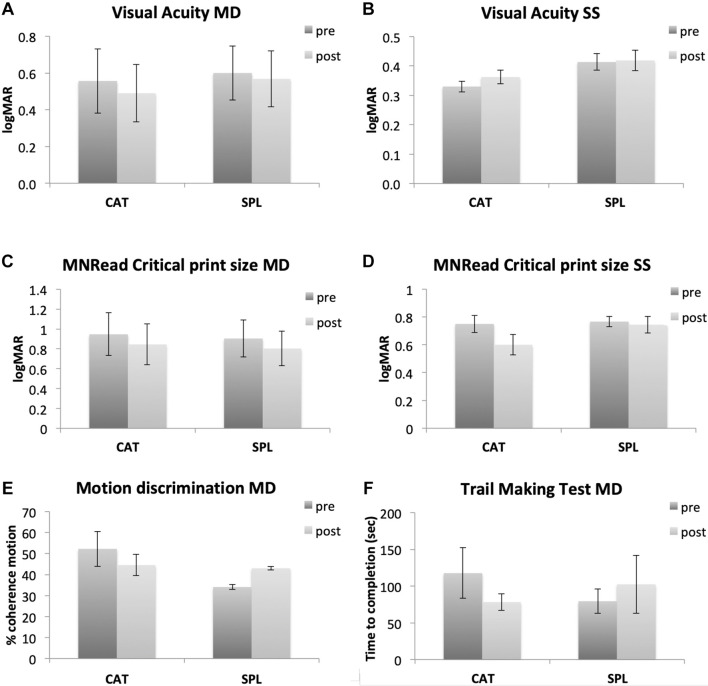
Preliminary results from our perceptual learning study comparing a standard perceptual learning (SPL) with a coordinated attentional training (CAT). In the first two rows, left side shows data from MD participants (**A,C** for visual acuity and MNRread respectively), while right side shows data from healthy participants trained with gaze-contingent, simulated scotoma (**B,D** for visual acuity and MNRread respectively). The last row shows additional assessment tasks only collected in MD participants (motion direction discrimination and Trail Making Test, **E,F** respectively). In SPL training, the Gabor target was always presented in the center of the screen and accompanied by a neutral acoustic cue. In CAT the target could appear anywhere on screen, thus involving visual search and gaze re-orienting. The target presentation was accompanied by a visual cue (a white circle around the target) and an auditory cue whose pitch and interaural difference were matched to the target location on screen. In the Trail Making Test, participants are presented with a piece of paper with circles containing numbers in random order and asked to use a pencil to connect them in ascending order as quickly as possible. The dependent measure of this test is the completion time expressed in seconds.

We note that while results are preliminary, they suggest that this CAT paradigm may lead to improvement in improvements in visual acuity (Figures 4A–D), and possibly also higher-level visual functions such as motion direction discrimination (Figure 4E) and in functional attention abilities measured by the Trail Making Test (Figure 4F). Additionally, the critical reading size measured with the MNRead showed a general improvement that was not specific for the training type. Future research will be required clarify both the most effective dosage of training, as some reports suggest that years of experience may be required certain changes in the visual system (Castaldi et al., 2016), as well as to understand how training may be differently effective as a function of age or disease etiology.

## Conclusion and Future Perspectives

Loss of central vision due to MD represents a serious health issue, with increasing incidence on the health system and the life of people worldwide (Wong et al., 2014). Patients suffering from central vision loss often adopt spontaneous coping strategies, including the development of a PRL, which replaces the fovea for tasks involving fixation, reading and fine detail vision. However, this solution is still suboptimal, and MD ends up taking a heavy toll on the quality of life of these patients. Currently, there are no definitive solutions to restore foveal vision, with most of the rehabilitative interventions focusing on optimizing the use of residual vision through visual training aimed at improving perceptual functions or oculomotor coordination.

Vision science and optometry, in particular, have tackled the issue of developing treatments to counteract the loss of central vision. These two fields adopt different approaches to rehabilitation. Specifically, vision science studies focus on perceptual improvements within the PRL, primarily in terms of contrast sensitivity, visual acuity and reading (Chung, 2011; Plank et al., 2014; Tarita-Nistor et al., 2014; Maniglia et al., 2016, 2020b), while optometry focuses on improving eye movement control (Nilsson et al., 2003; Coco-Martin et al., 2013; Rosengarth et al., 2013). Both approaches have their merits, since both visual abilities and oculomotor coordination within the PRL are usually poor in MD patients; however, interventions stemming from these fields seem to encounter variable degree of success, and no one-fits-all solution appears available.

A further complication emerges when considering spontaneous compensatory oculomotor strategies, and the process of saccadic re-referencing, in which the PRL becomes the new reference spot for ballistic eye movements (White and Bedell, 1990). This process can take up to several months and, in some cases, might still remain incomplete (Fletcher and Schuchard, 1997; Crossland et al., 2005). The mechanisms involved in the selection and development of the PRL are still unclear, with studies using a simulated scotoma in healthy participants suggesting that attentional resources might play a role in its selection (Barraza-Bernal et al., 2017). Indeed, the use of eye tracker-guided simulated scotomas as a framework for the study of the development of eye movement strategies in conditions of simulated central vision loss represents an exciting perspective toward understanding these mechanisms. The gaze-contingent simulation of central vision loss allows for a tightly controlled setup in which several parameters (onset, size of the scotoma, time of exposure to central vision loss, etc.) can be modified and compared, while avoiding common drawbacks of clinical research such as difficulty in recruitment and poor compliance. Encouraging results show that some of the oculomotor behavior observed in patients, such as PRL development, can be reproduced with the simulated scotoma paradigm, although with differences in the time course (Kwon et al., 2013; Walsh and Liu, 2014; Barraza-Bernal et al., 2017a,b; Chen et al., 2019; Maniglia et al., 2020a). Still, it is unclear the extent to which these simulated scotoma approaches, which typically involve just days to weeks of exposure, compare to the longer-term experience that patients have with central vision loss. While training, and hard-boundaries of the simulated scotoma, which provide awareness of the scotoma, may explain some component of the rapid plasticity seen with simulated scotomas, other work, such as that with retinal implants (Castaldi et al., 2016), suggests that plasticity can continue for years and that there are likely longer time course aspects of plasticity that remain to be clarified.

Finally, a more thorough investigation of eye movements, which takes into account temporal aspects of fixation strategies and both within- and between-trial behaviors, might shed light on the mechanisms underlying the development of compensatory oculomotor strategies and guide individualized intervention (e.g., Crossland et al., 2005; Maniglia et al., 2019).

We suggest that to develop effective interventions, it is necessary to approach MD from a different standpoint that takes into account the larger consequences of central vision loss, which encompass basic visual functions and oculomotor coordination and also affect cognitive and attentional mechanisms. Our preliminary data suggest the potential benefit of an integrated intervention in the form of a visuo-attentional training that aims at engaging these three components (low-level vision, oculomotor system and attentional networks) simultaneously to promote functional brain plasticity. We encourage additional research in this direction both to determine the extent to which this integrated model can aid those with central vision and also to further determine the mechanism by which different types of training give rise to plasticity in those experiencing central vision loss.

## Data Availability Statement

The raw data supporting the conclusions of this article will be made available by the authors, without undue reservation.

## Ethics Statement

The studies involving human participants were reviewed and approved by University of California, Riverside. The patients/participants provided their written informed consent to participate in this study.

## Author Contributions

MM collected and analyzed the data for the training study and wrote the first draft of the manuscript. All authors contributed to conception of the manuscript, the design of the training study, data interpretation, and manuscript revision, read, and approved the submitted version.

## Conflict of Interest

The authors declare that the research was conducted in the absence of any commercial or financial relationships that could be construed as a potential conflict of interest.

## Publisher’s Note

All claims expressed in this article are solely those of the authors and do not necessarily represent those of their affiliated organizations, or those of the publisher, the editors and the reviewers. Any product that may be evaluated in this article, or claim that may be made by its manufacturer, is not guaranteed or endorsed by the publisher.
