# Supplementary material for: Perspective on Vision Science-Informed Interventions for Central Vision Loss
Source: Front Neurosci. 2021 Nov 4;15:734970. doi: 10.3389/fnins.2021.734970 (PMC8599964; doi:10.3389/fnins.2021.734970)
Supplement: Supplementary file 1 [file Table_1.DOCX]

|  | **Training effect overall** | **Training effect CAT** | **Training effect SPL** | **Training type x Training effect** |
| --- | --- | --- | --- | --- |
| **Visual Acuity SS** | F(1,17)=0.656, p=0.429, η^2^= 0.012 Bayes factor= 0.425 | t(9)=1.01, p= 0.830 | t(8)=0.162, p= 0.562 | F(1,17)=0.331, p=0.573, η^2^= 0.006 Bayes factor= 0.948 |
| **Visual Acuity MD** | *F(1,9)=7.067, p=0.026, η^2^= 0.005, Bayes factor= 2.826* | *t(5)=2.654, p= 0.023* | t(4)=1.173, p= 0.153 | F(1,9)=0.872, p=0.375, η^2^= 0.001, Bayes factor=2.507 |
| **Critical Print Size MD** | F(1,9)=3.37, p=0.1, η^2^= 0.014, Bayes factor= 1.151 | t(5)=1.146, p= 0.152 | t(4)=1.826, p= 0.071 | F(1,9)=0.001, p=0.988, η^2^= 0.001, Bayes factor = 0.882 |
| **Critical Print Size SS** | F(1,17)=2.91, p=0.1, η^2^= 0.052, Bayes factor= 1.119 | t(9)=2.087, p=0.067 | t(8)=0.316, p=0.38 | F(1,17)=1.61, p=0.223, η^2^= 0.028, Bayes factor = 0.668 |
| **Motion Discrimination MD** | F(1,9)=0.012, p=0.914, η^2^= 0001., Bayes factor=0.375 | t(5)=1.273, p= 0.129 | t(4)=0.949, p= 0.802 | F(1,9)=2.368, p=0.158, η^2^= 0.036, Bayes factor=0.269 |
| **TMT-b MD** | F(1,9)=0.21, p=0.657, η^2^  = 0.005, Bayes factor=0.429 | t(5)=1.53, p= 0.092 | t(4)=0.941, p= 0.800 | F(1,9)=3.027, p=0.116, η^2^= 0.065, Bayes factor=0.236 |
| **VA (PRL Test) SS** | *F(1,17)=7.265, p=0.015,* η^2^*= 0.168 Bayes factor= 7.284* | t(9)=1.58, p=0.074 | t(8)=2.156, p=0.032 | F(1,17)=2.118, p=0.164, η^2^= 0.049 Bayes factor= 3.419 |

**Table 1**. Statistical comparisons for assessment tasks measured before and after CAT or SPL training in participants with macular degeneration (MD) and healthy participants trained with simulated scotoma (SS, see main manuscript for details). Training effect overall and Training type x Training effect analyses conducted with repeated measure ANOVAs with Time (pre vs post) as within factor and Group (CAT vs SPL) as between factor. All training effect analyses within each training type conducted as one-tailed, paired t-tests.
